# Supplementary material for: Conductance quantization suppression in the quantum Hall regime
Source: Nat Commun. 2018 Feb 13;9:659. doi: 10.1038/s41467-018-03064-8 (PMC5811439; doi:10.1038/s41467-018-03064-8)
Supplement: Supplementary file 1 — Supplementary Information [file 41467_2018_3064_MOESM1_ESM.pdf]

Supplementary Information

Conductance quantization suppression in the quantum Hall regime

José M. Caridad[1*†], Stephen R. Power[1,2,3*], Mikkel R. Lotz[1], Artsem A. Shylau[1], Joachim D. Thomsen[1], Lene Gammelgaard[1], Timothy J. Booth[1], Antti-Pekka Jauho[1], Peter Bøggild[1†]

[1] *Centre for Nanostructured Graphene (CNG), Department of Micro- and Nanotechnology, Technical University of Denmark, 2800 Kongens Lyngby, Denmark*

[2] *Catalan Institute of Nanoscience and Nanotechnology (ICN2), CSIC and The Barcelona Institute of Science and Technology, Campus UAB, Bellaterra, 08193 Barcelona, Spain*

[3] *Universitat Autònoma de Barcelona, 08193 Bellaterra (Cerdanyola del Vallès), Spain*

*These authors contributed equally to this work

[†] corresponding authors: jcar@nanotech.dtu.dk, peter.boggild@nanotech.dtu.dk

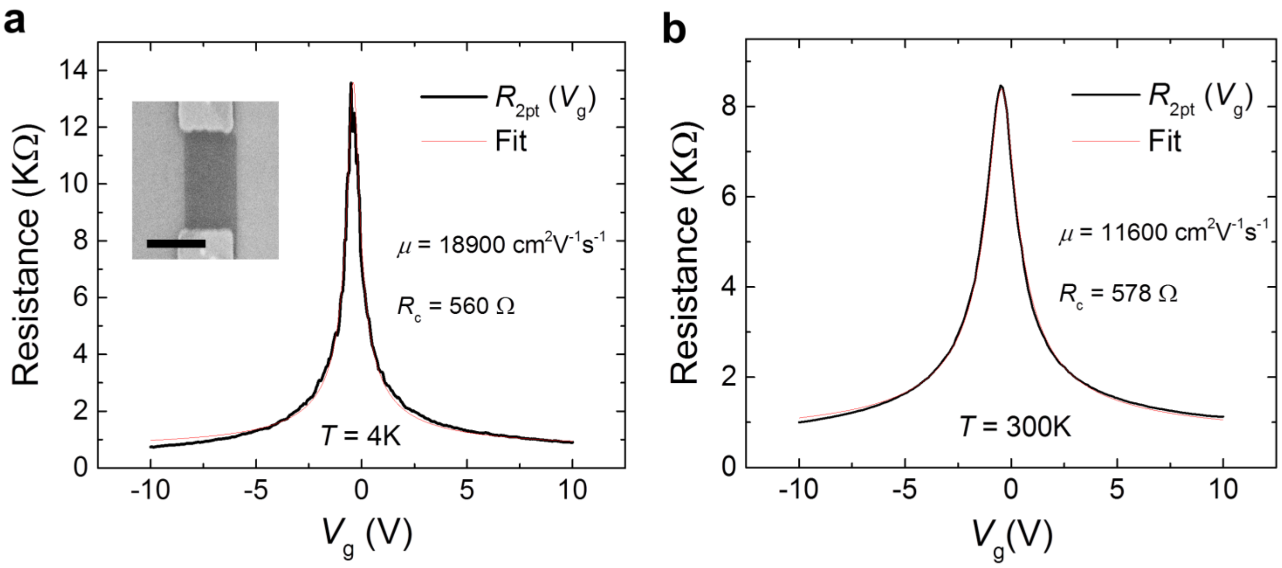

Supplementary Figure 1. Resistance of pre-constriction devices (graphene strips of width W = 1 μm) at different temperatures. (**a**) Two-terminal (2-pt) resistance (R_{2pt}) of a device at a temperature T = 4 K. Fitting the experimental data with Supplementary Equation (1), we extract a μ = 18900 cm^{2}V^{-1}s^{-1} and a R_c = 560 Ω. Inset shows a scanning electron micrograph of this graphene device. Scale bar is 1 μm. (**b**) 2-pt resistance of the same device at T = 300 K. Fitting the experimental data with Supplementary Equation (1), we obtain a μ = 11600 cm^{2}V^{-1}s^{-1} and a R_c = 578 Ω.

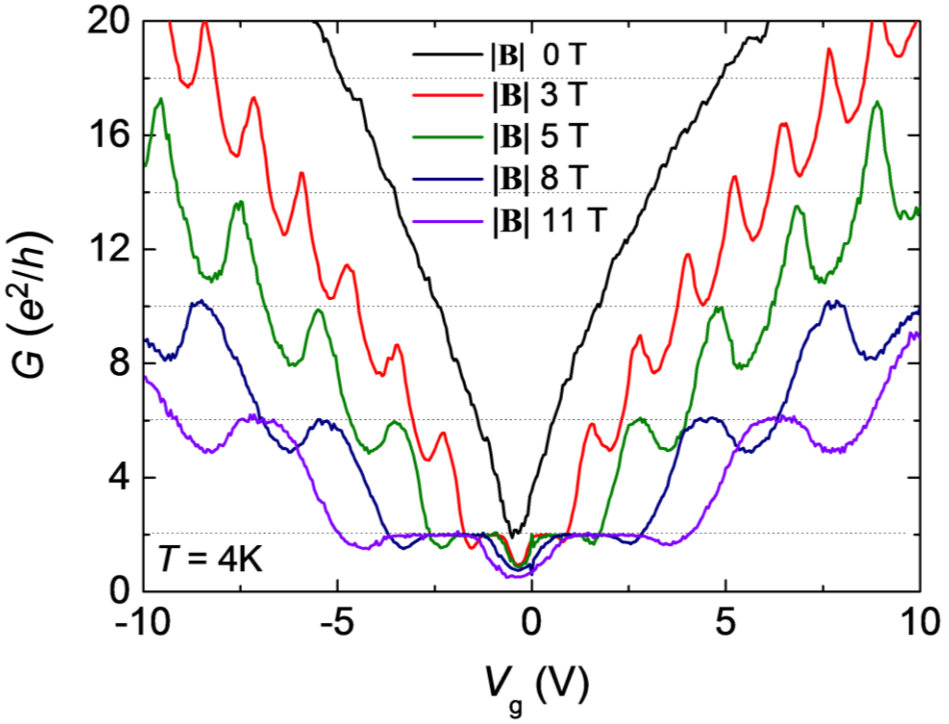

Supplementary Figure 2. Magnetotransport characteristics of initial pre-constriction devices (graphene strips of width $W = 1$ μm) at $T = 4$ K. Magnetoconductance of the device shown in Supplementary Fig. 1 at $T = 4$ K, showing integer quantum Hall (QH) plateaus at filling factors $N = \pm 2, \pm 6, \pm 10, \ldots$ as expected for single layer graphene. The contact resistance R_c 510 Ω was subtracted from the R_{2pt} data.

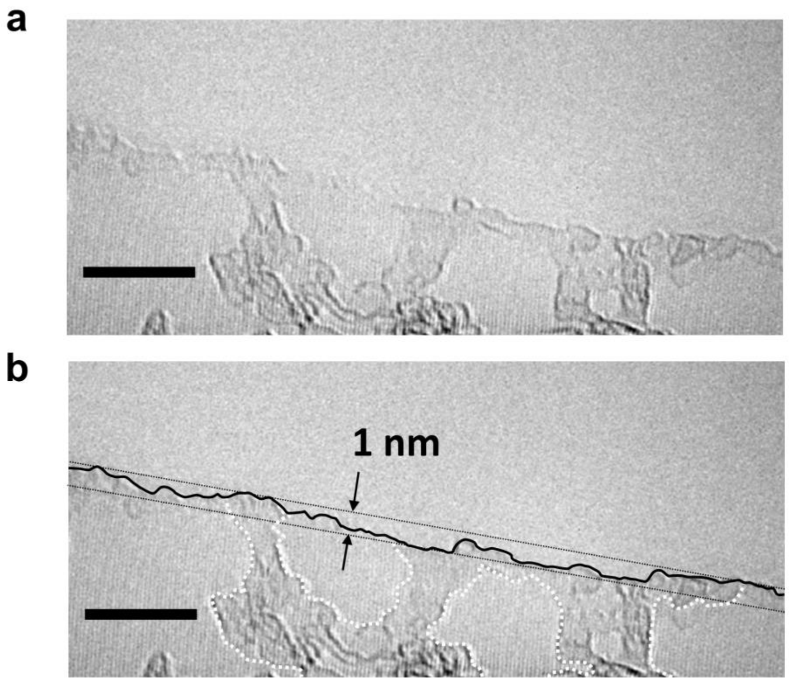

Supplementary Figure 3. Transmission electron micrograph (TEM) showing an etched graphene border. This sample was etched using reactive ion etching, the utilized procedure for our Sample type 1 devices and shows an edge roughness $\leq 1\,\mathrm{nm}$. **(a)** TEM image. Scale bar is 5 nm. **(b)** Same TEM image indicating (for clarity) the graphene border (continuous black line) and the 1 nm width containing the graphene border (dotted black line). Dotted white lines delimit areas with PMMA residues resulting from the electron beam lithography step or CAB residues from the transfer step to the TEM grid. Scale bar is 5 nm.

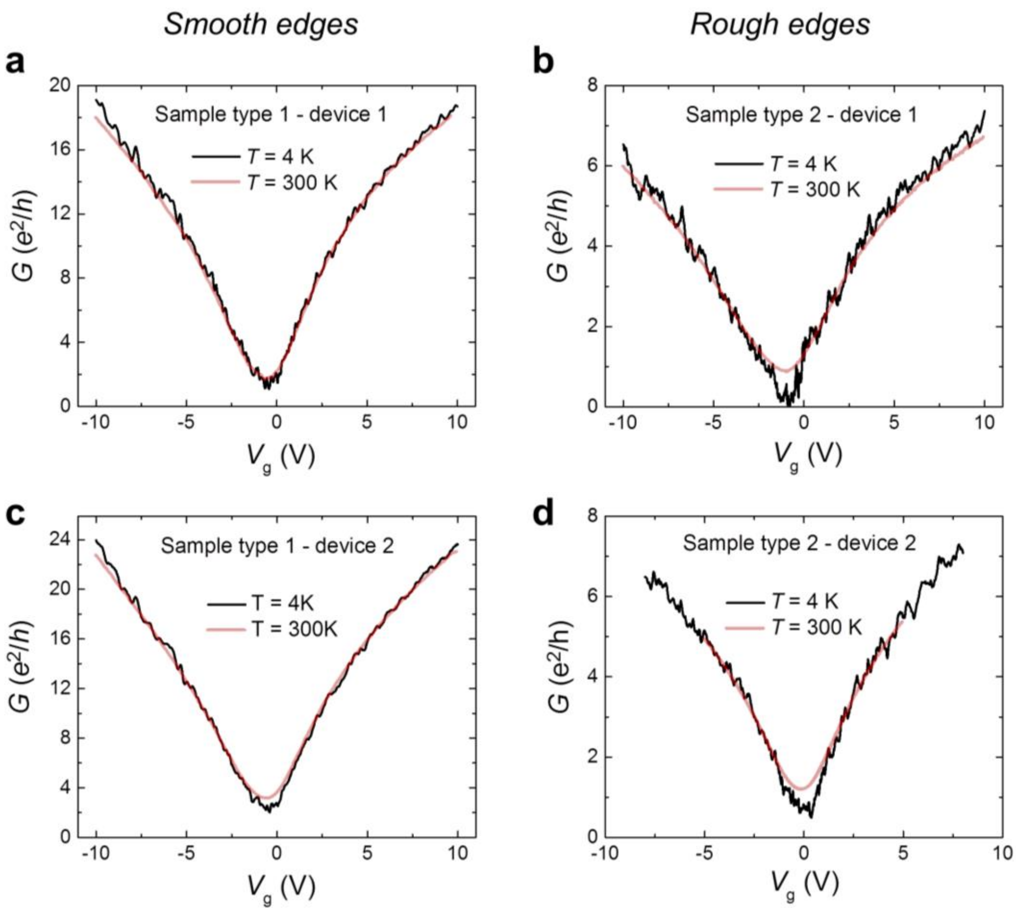

Supplementary Figure 4. Conductance measurements in graphene nanoconstrictions at *T* = 4 K and *T* = 300 K for $|\mathbf{B}|$ = 0 T. (a) Conductance G as a function of the gate voltage V_g in a graphene nanoconstriction with low edge roughness (Sample type 1, Device 1, see Fig. 2a, main text) at T = 4 K (black) and T = 300 K (translucent red). The extracted R_c in this sample is 410 Ω. (**b)** Conductance G as a function of the gate voltage V_g in a graphene nanoconstriction with large edge roughness (Sample type 2, Device 1, see Fig. 2b, main text) at T = 4 K (black) and T = 300 K (red). The extracted R_c in this sample is 518 Ω. (**c)** and **(d),** shows similar data as in (**a)** and (**b**), respectively, but measured in a second set of devices of both sample types. The extracted R_c in these samples are 560 Ω and 590 Ω, respectively.

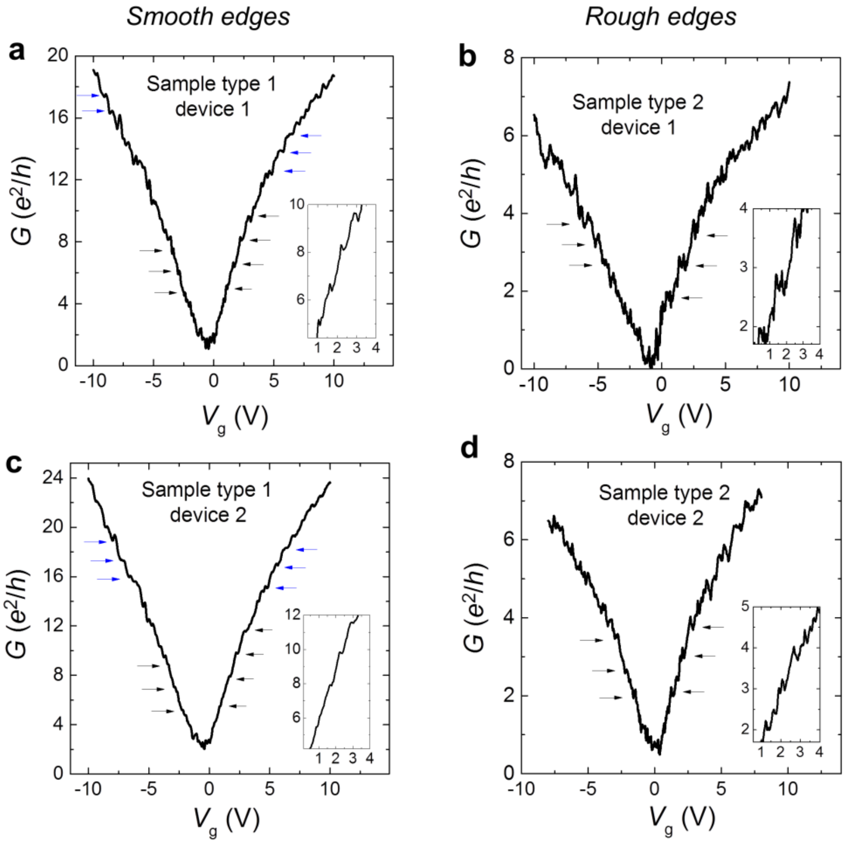

Supplementary Figure 5. Size quantization measurements in graphene nanoconstrictions in samples with different type of edge roughness (Sample types 1 and 2) at *T* = 4 K and $|\mathbf{B}|$ = 0 T. **(a),** Sample type 1, device 1, same device as in Supplementary Fig. 4a. Inset shows periodic conductance modulations with step heights up to $\Delta G \sim 2e^2/h$. **(b),** Sample type 2, device 1, same device as in Supplementary Fig. 4b . Inset shows periodic conductance modulations with step heights up to $\Delta G \sim e^2/h$.

(c), Sample type 1, device 2, same device as in Supplementary Fig. 4c. Inset shows periodic conductance modulations with step heights up to $\Delta G \sim 2e^2/h$. **(d),** Sample type 2, device 2, same device as in Supplementary Fig. 4d. Inset shows periodic conductance modulations with step heights up to $\Delta G \sim e^2/h$. The extracted R_c in these samples are shown in the caption of Supplementary Fig. 4.

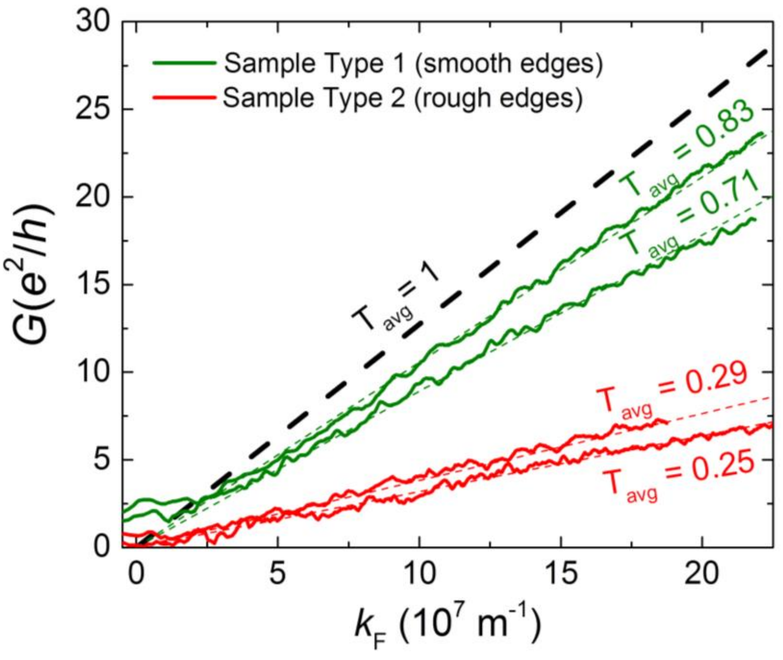

Supplementary Figure 6. Average transmission coefficient T_{avg} in graphene nanoconstrictions in samples with different type of edge roughness (Sample types 1 and 2) at T = 4 K and $|\mathbf{B}|$ = 0 T. Black dashed line indicates a unit transmission coefficient for a channel of width W = 100 nm.

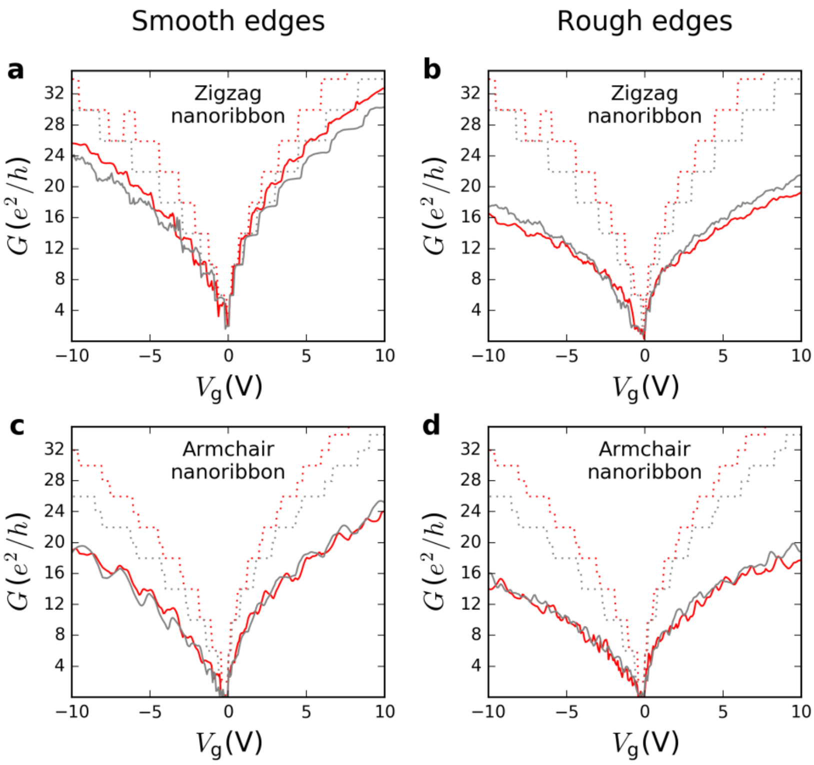

Supplementary Figure 7. Conductance simulations at $|\mathbf{B}| = 0$ T for pristine (dotted) and smooth **(a,c)** and rough **(b,d)** edge disordered nanoribbons with both zigzag **(a,b)** and armchair **(c,d)** edge geometries. Similar features are noted for both constant (grey) and non-uniform (red) gating approaches.

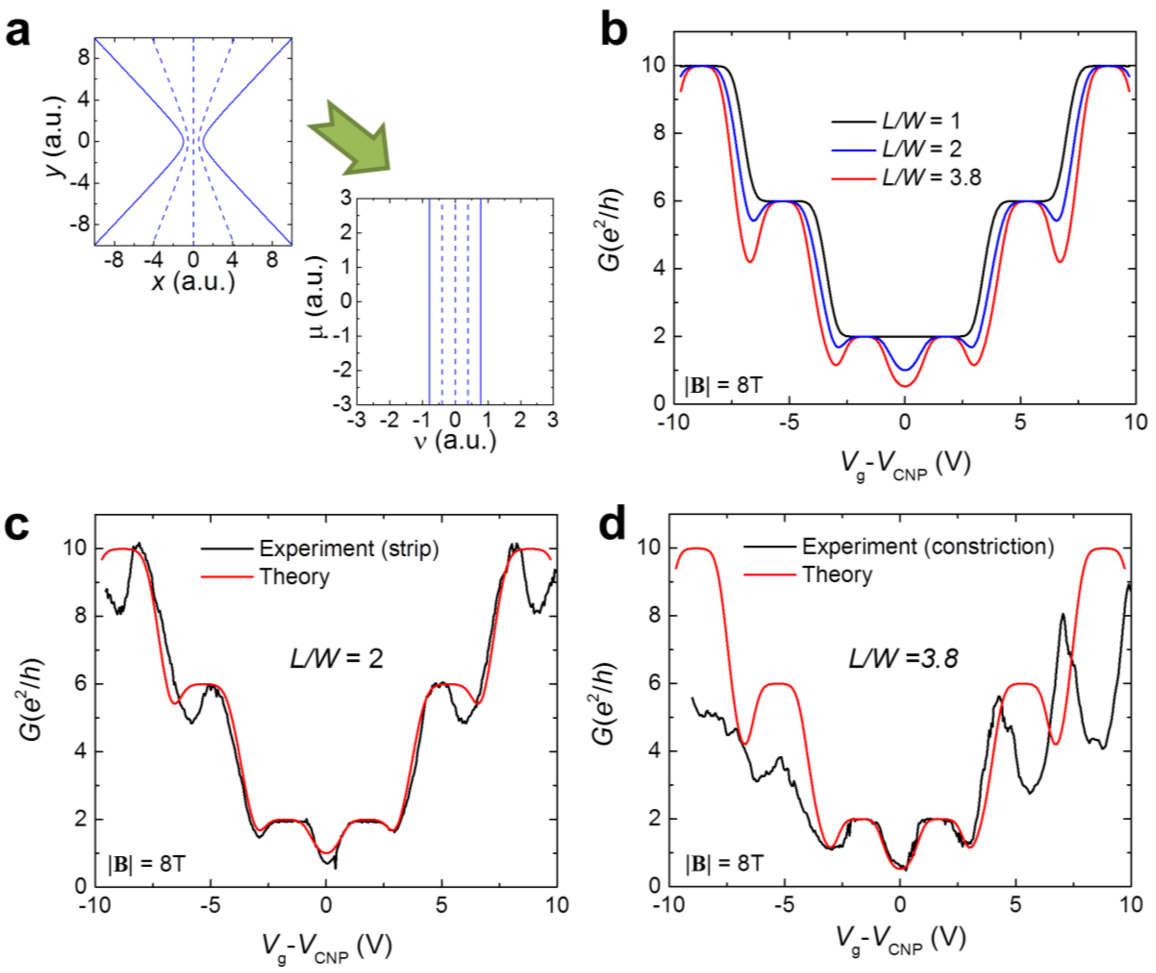

Supplementary Figure 8. Geometrical corrections for the quantum Hall conductance in homogeneous two-terminal graphene nanoconstrictions. (a) Full constriction geometry mapped onto an equivalent rectangle of aspect ratio $L/W \sim 3.8$. **(b)** Two-terminal conductance calculation for rectangles with different L/W at $|\mathbf{B}| = 8$ T. **(c)** Comparison between experimental and calculated conductance for rectangles with $L/W \sim 2$ (graphene strips, Supplementary Fig.2) at $|\mathbf{B}| = 8$ T. **(d)** Comparison between experimental and calculated conductance for rectangles with $L/W \sim 3.8$. The experimental data is the one of constrictions with rough edges (Sample type 2, Fig. 2b, main text at $|\mathbf{B}| = 8$ T), where no CQS effect is observed. The extracted λ (see Supplementary Note 4) in panels **(c)** and **(d)** is ~2.

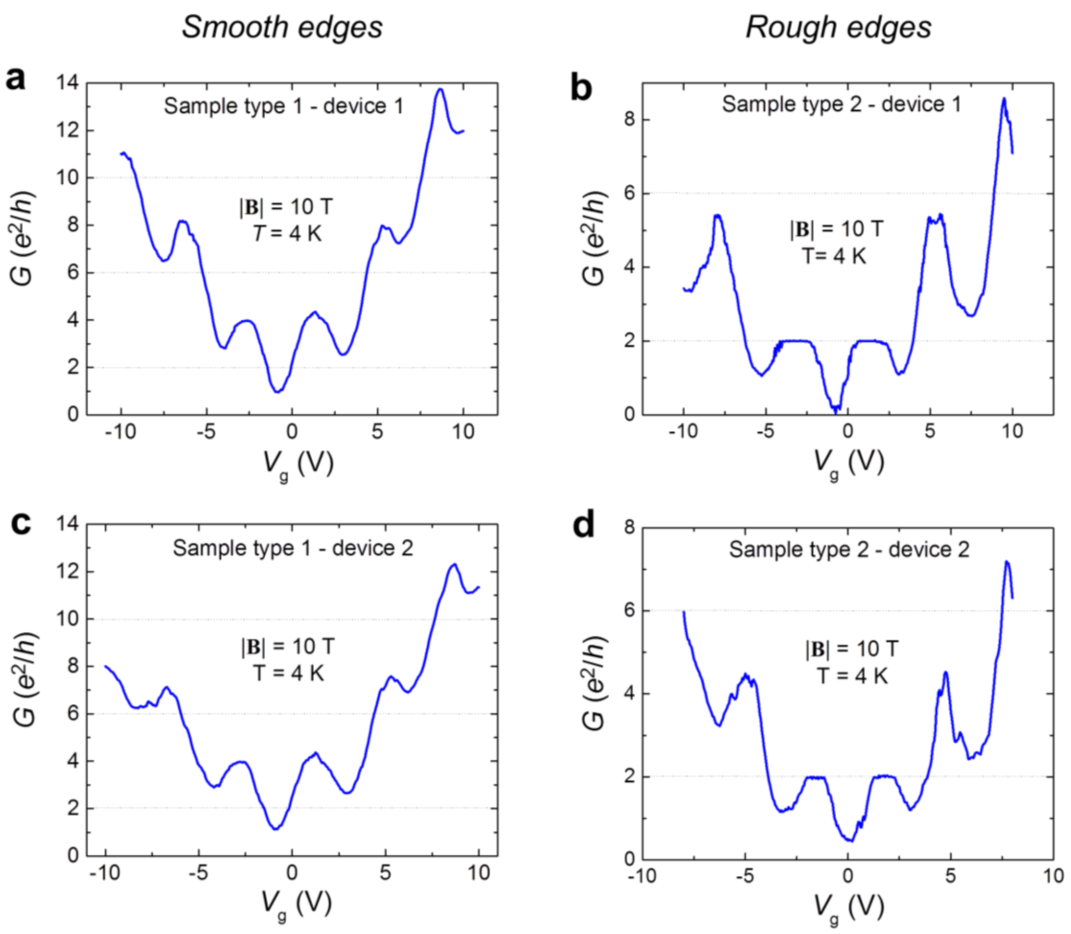

Supplementary Figure 9. Conductance measurements in graphene nanoconstrictions (W = 100 nm, L = 100 nm) at T = 4 K and $|\mathbf{B}|$ =10 T. Devices with low edge roughness (Sample type 1, **(a)** and **(c)**) show a non-quantized magnetoconductance with values higher than the single-electron picture (CQS effect). This phenomenon does not occur in devices with larger edge roughness (Sample type 2, **(b)** and **(d)**) where quantization is exhibited at $G = 2e^2/h$ (LL0). Dotted lines show conductance values where, according to the single-electron picture, a plateau is expected to appear in a graphene device in the QH regime (filling factors ± 2, ± 6, ± 10,…). The extracted R_c in these samples are shown in the caption of Supplementary Fig. 4.

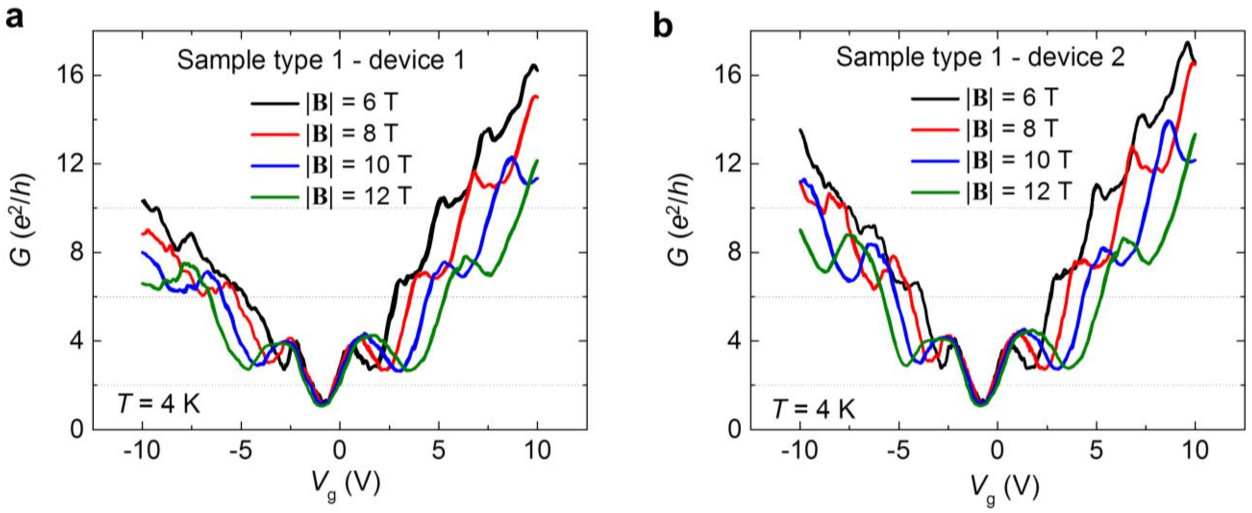

Supplementary Figure 10. **Conductance measurements in graphene nanoconstrictions (*W* = 100 nm, *L* = 100 nm) with low edge roughness (Sample type 1) at *T* = 4 K from $|\mathbf{B}|$ = 6 T to $|\mathbf{B}|$ = 10 T**. **(a)** Device 1, previously introduced in Supplementary Fig. 4a. **(b)** Device 2, previously introduced in Supplementary Fig. 4c. Both devices show a clear non-quantized magnetoconductance (CQS effect) for all the magnetic fields. The extracted R_c in these devices are shown in the caption of Supplementary Fig. 4. Dotted lines in both panels show conductance values where a plateau is expected to appear in a graphene device in the QH regime according to the single-electron picture.

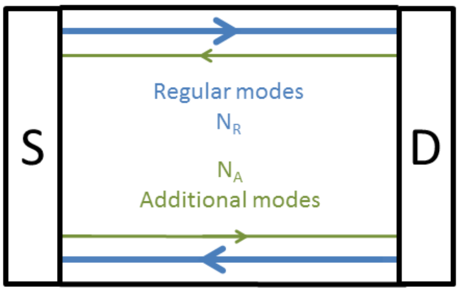

Supplementary Figure 11. Additional counter-propagating edge states. Illustration of counter-propagating edge states: regular (blue) and additional (green). Additional edge states due to a large charge accumulation at edges of narrow, ballistic and hard-wall confined two-terminal devices.

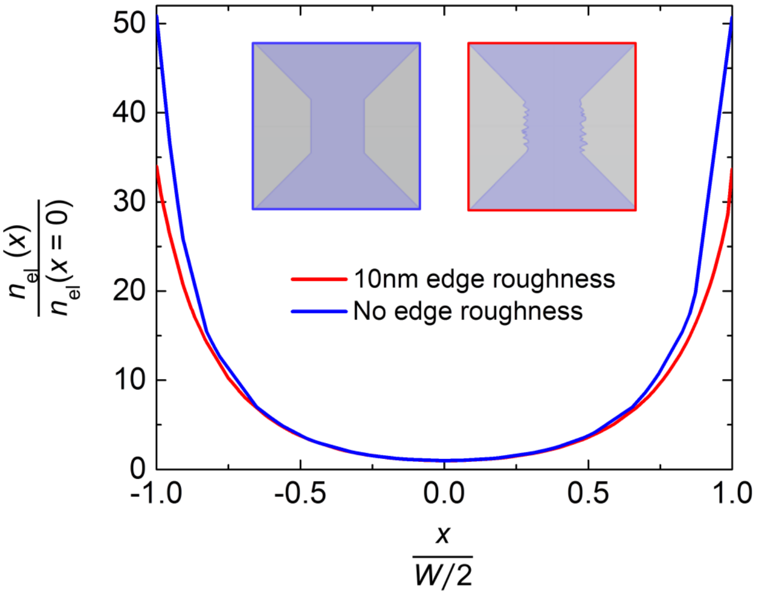

Supplementary Figure 12. **Edge roughness effect on the carrier density accumulation across graphene nanoconstrictions: electrostatic approach.** Having similar dimensions (W = 100 nm, L = 100 nm), devices with larger edge roughness have less charge accumulation close to the borders.

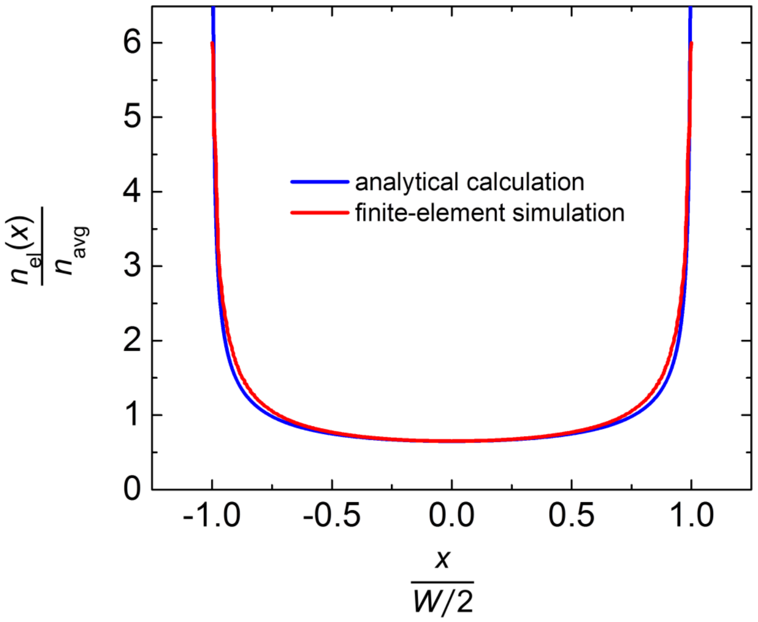

Supplementary Figure 13. **Comparison of $\frac{n_{\mathrm{el}}(x)}{n_{\mathrm{avg}}}$ between analytical calculation[23] and the finite-element method simulation (Methods) for infinitely long graphene ribbons with widths $W = 100$ nm.** Analytical and computational calculations show an excellent agreement, even when only accounting for the first (main) term of the analytical calculation[23].

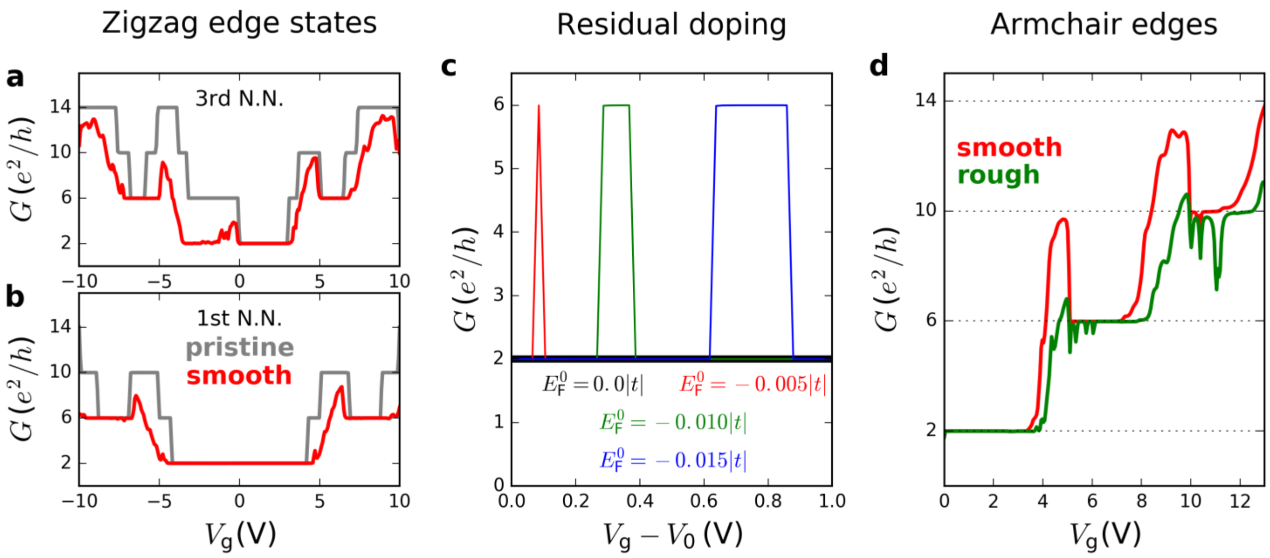

Supplementary Figure 14. **Additional simulation features at $|\mathbf{B}|$ = 10 T**. **(a)**, An electron hole asymmetry arises for pristine ribbons due to dispersive zigzag edge states near the CNP within the 3rd nearest neighbor tight binding model. This tends to vanish for even smooth edge disorder and is not present for **(b)** the 1st nearest neighbor model where such states are non-dispersive. **(c)**, A conductance peak corresponding to a bending of LL0 only occurs when the charge neutrality and zero gating points are shifted relative to each other – these results show the emergence of such a feature for an increased residual charge at zero gate in a zigzag ribbon within the 1st nearest neighbor model. **(d)**, The CQS peaks are not strongly dependent on edge geometry, and are shown to emerge also in armchair edged ribbons with smooth disorder, but are suppressed when strong disorder is present as in the zigzag case.

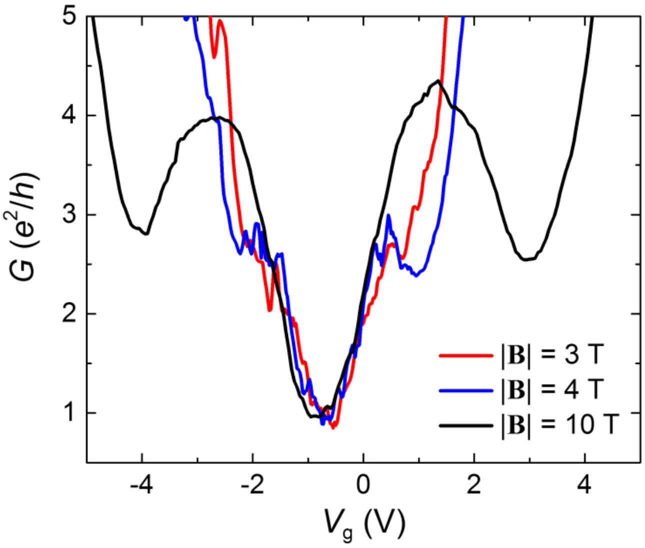

Supplementary Figure 15. **CQS effect is observed in smooth graphene nanoconstrictions at low magnetic field.** The CQS effects starts to be observable from $|\mathbf{B}|$ = 3-4 T in our graphene constrictions with smooth edges (Sample type 1) for the LL0.

Supplementary Note 1. Device quality: Graphene mobility and contact resistance

Graphene strips of widths $W = 1$ µm (see Supplementary Fig. 1a, inset) were measured to estimate the bulk mobility μ (thus, mean free path, l_{mfp}) of our graphene flakes and the contact resistance R_{c} of the devices prior to the definition of the actual nanoconstrictions. We use a two parameter fitting to extract the average mobility μ and R_{c} values[1,2] at a given temperature T :

$$R_{2\mathrm{pt}} = R_{\mathrm{c}} + \frac{L/W}{e\mu\sqrt{n^2 + n_{\mathrm{res}}^2}} \quad (1).$$

Here, $R_{2\mathrm{pt}}$ is the measured two-point resistance of the device, e is the elementary charge, n_{res} is the residual carrier concentration and n is the back-gate induced carrier concentration given by:

$$n = \frac{C_{\mathrm{ox}}}{e}\left(V_{\mathrm{g}} - V_{\mathrm{CNP}}\right), \quad (2),$$

where C_{ox} is the gate oxide capacitance per unit area for an infinite plane capacitor (3.5×10^{-4} Fm^{-2} for 100 nm thick SiO_2) and V_{CNP} is the position of the charge neutrality point (CNP).

Our devices show (Supplementary Fig.1a) a relatively low disorder density (inhomogeneities) at $T = 4$ K. Their resistivity $\rho(V_{\mathrm{g}})$ full-width at half-maximum FWHM is < 1V, with $\rho(V_{\mathrm{g}})$ calculated as $\rho(V_{\mathrm{g}}) = (R_{2\mathrm{pt}} - R_{\mathrm{c}})(W/L)$. This FWHM establishes an upper bound of disorder-induced carrier density fluctuations of[3] $\delta n < 2\times10^{11}$ cm^{-2}. The position of the charge neutrality point is <0.5 V indicating a relatively small level of residual charge-carriers $n_{\mathrm{res}} \sim 1\times10^{11}$ cm^{-2} and the field-effect mobility μ is around ~20000 $\mathrm{cm}^2\mathrm{V}^{-1}\mathrm{s}^{-1}$ for both carrier types at low temperatures ($T = 4$ K). We note that these mobility values agree with those reported in literature for similarly prepared graphene samples [4]. The semiclassical mean free path l_{mfp} for

these devices is $l_{\mathrm{mfp}} = \frac{\mu h}{2e}\sqrt{\frac{n}{\pi}}$ ~ 215 nm at a typical carrier density of n = 1×10^{12} cm^{-2}. This estimated value is well above the feature size of our nanoconstrictions (~100 nm), ensuring that the transport is limited by boundary scattering[5] in the latter etched devices. Supplementary Fig. 1b shows the dependence $R_{2\mathrm{pt}}(V_{\mathrm{g}})$ of the graphene strips at room temperature, exhibiting a decreased mobility to ~12000 $\mathrm{cm}^2\mathrm{V}^{-1}\mathrm{s}^{-1}$ due to the role of electron-phonon scattering[3,5]. We note that the variation of extracted contact resistance ΔR_{c} is below 50 Ω from T = 300 K to T = 4 K (see Supplementary Fig.1) .

Moreover, we confirmed μ and R_{c} values in these graphene strips by undertaking magnetoconductance measurements. Supplementary Fig. 2 shows the two-point conductance $G = (R_{2\mathrm{pt}} - R_{\mathrm{c}})^{-1}$ as a function of the back-gate V_{g} and perpendicular magnetic fields **B** of different strengths at T = 4 K. Integer quantum Hall (IQH) plateaus at filling factors N = ± 2, ± 6, ± 10 appear as expected for single layer graphene [6,7]. We note that the development of the plateau at N = ± 2 starts to be visible from $|\mathbf{B}|$ > 1 T (not shown); agreeing with the measured μ ~20000 $\mathrm{cm}^2\mathrm{V}^{-1}\mathrm{s}^{-1}$ (assuming $\mu|\mathbf{B}|$>>1 for the IQH to exist). Furthermore, to coincide with the expected plateau values, the subtracted R_{c} in Supplementary Fig. 2 was 510 **Ω**, in good agreement with the value extracted by fitting the experimental data in Supplementary Fig. 1 with Supplementary Equation (1) (~560 **Ω**). Lastly, the quantized magnetoconductance exhibits a minimum after a plateau when going towards higher gate voltages. This behavior agrees with geometrical effects in homogeneous graphene samples with L/W >1 measured in a 2-terminal configuration due to the mixing of longitudinal and transverse resistivities[7] (see Supplementary Note 4).

Supplementary Note 2. Assessment of edge roughness in graphene nanoconstrictions.

Edge disorder plays a key role in the transport characteristics of narrow graphene devices[8-12]. In particular, the conductance quantization suppression (CQS) phenomenon reported in this work is highly dependent on the achievement of a low level of edge roughness in experimental devices. CQS appears in our devices etched with Ar/O_2 reactive ion etching, RIE (Sample type 1) an etching procedure with introduces much less edge disorder than O_2 plasma ashing (Sample type 2).[12]

We assess the amount of edge roughness in type 1 devices using transmission electron microscopy (TEM). This structural information allows us to carry out realistic tight-binding simulations with similar levels of disorder (Methods) in order to evaluate the persistence of the CQS effect in such samples.

Our TEM images were recorded as follows. First, graphene flakes were exfoliated on Si/SiO_2 wafers and transferred to DuraSiN™ TEM grids. To do so, the crystals are detached from the substrate through the use of a hydrophobic polymer, cellulose acetate butyrate (CAB), and water. By spin coating the Si/SiO_2 substrate with CAB, the hydrophobic/hydrophilic interaction of CAB/ SiO_2 will allow the water to intercalate, leaving the graphene attached to the released CAB film. The polymer with attached graphene is transferred manually to the TEM sample carrier and then dissolved in acetone. The sample is rinsed in isopropyl alcohol and then dried using a critical point dryer. Once graphene flakes are on TEM grids, we performed electron beam lithography (EBL) and Ar/O_2 RIE etching of these graphene samples in a similar way to

the supported nanoconstrictions (Methods). The PMMA resist layer was removed by immersing the sample in acetone (5 mins), isopropanol (1min) and drying it again using a critical point dryer. Finally, samples were imaged using a spherical aberration corrected FEI Titan TEM equipped with a monochromator operated at 80 keV. Supplementary Fig. 3 shows one of our TEM images along a graphene edge. The edge roughness is below 1nm, a value that is comparable graphene nanoribbons having very low edge roughness, fabricated by unzipping carbon nanotubes[11].

Supplementary Note 3. Further devices: Conductance at $|\mathbf{B}|$ = 0 T in graphene nanoconstrictions with inhomogeneous charge density

The conductance at zero **B** (Figs. 2a,2b, main text) in both types of etched nanoconstrictions (Sample type 1 and 2) show a $G \propto (\Delta V_g)^{1/2}$ behavior, characteristic of transport limited by boundary scattering [5,13,14]. Supplementary Figs. 4a-d corroborate this observation, showing how G values in constrictions defined with both types of edge disorder have no significant temperature dependence of from 4 K to 300 K away from the charge neutrality point, CNP (gate voltages $|V_g - V_{CNP}|$ >1 V). We note that this fact cannot be due to charge impurities in the graphene channel since the electrical bulk-flake behavior does depend on temperature (Supplementary Fig. 1).

There are clear differences between Sample type 1 and Sample type 2 devices. The values of $G = G(V_g, |\mathbf{B}|)$ in Sample type 1 (Supplementary Figs. 4a,c) are much larger (×3 times) than those for Sample type 2 (Supplementary Figs. 4b,d). This effect is consistent with the role of different degrees of edge disorder in the two type of samples[10-12]: Sample type 1 is etched with Ar/O_2 reactive-ion-etch, a procedure that produces less disorder than O_2 plasma ashing [12], the etching technique used in the case of Sample type 2. The effect of a lower edge disorder present in Sample type 1 can be further seen in Supplementary Figs. 3a,b, at positions close to the CNP ($|V_g - V_{CNP}|$ < 1 V). G in these devices has similar values at T = 300 K and 4 K, all above $2e^2/h$. This is not the case of constrictions with higher edge disorder (Sample type 2, Supplementary Figs. S4b,d). The latter samples present a conductance which tends to zero close to the CNP at T = 4 K; a well-known effect due to the appearance of a transport gap in narrow and disordered graphene devices[8,15].

Additionally, at $T = 4$ K, both type of samples (Supplementary Figs. 5a,b) exhibit clear periodic modulations (conductance kinks, see Supplementary Figs. 5 Insets), an indication of size quantization[13]. The step height ΔG of these modulations is up to ~$2\,e^2/h$ in the case of Sample type 1 (Supplementary Figs. 5a,c). Meanwhile, conductance modulations only up to $\Delta G \sim e^2/h$ appear in our nanoconstrictions with rougher edges (Sample type 2, Supplementary Figs. 5b,d). Conductance kinks in Sample type 2 are less obvious due to the presence of more, rapidly varying aperiodic fluctuations in their electrical characteristics with respect to the gate voltage, an effect linked to edge disorder [12]. We note that these aperiodic conductance fluctuations in both types of samples have approximately a magnitude of ~$0.5\,e^2/h$, compatible with Universal Conductance Fluctuations (UCF) [16] .This reasoning is further indicated by the absence of such fluctuations at high magnetic fields.

In order to compare further the quality of both types of etched nanoconstrictions, we have estimated the averaged transmission coefficient $0 < T_{\text{avg}} < 1$ of our constrictions by taking into account the semi-classical relation[13] $G = \dfrac{4e^2}{h} k_{\text{F}} T_{\text{avg}} \dfrac{W}{\pi}$.

For this calculation, we take $k_{\text{F}} = \sqrt{\pi n_0}$ where n_0 is the charge density calculated for an infinite capacitor. We note that this is an approximation since the averaged charge density n_{avg} of nanostructures with inhomogeneous charge density $n(x)$ is larger than n_0. For example, in the case of a graphene strip, $n_{\text{avg}} = n_0\,\pi/2$ (Methods). Supplementary Fig. 6 shows the averaged transmission of our devices. Within our approximation, nanoconstrictions of type 1 (smooth edges) have a transmission coefficient larger than 0.7, meanwhile nanoconstrictions of type 2

(rough edges) have a transmission coefficient below 0.3, confirming the fact that edge roughness plays a key role in the charge transport of narrow graphene devices.

Lastly, we note that the periodic conductance kinks in Sample type 1 (Supplementary Fig. 5) tend to be smaller (blue arrows) and disappear towards higher gate voltages. This behaviour agrees with theoretical calculations of coherent transport in graphene nanoribbons with moderate edge disorder[8,17]. It is explained by the fact that edge-defect scattering occurs equally in all the subbands, resulting in a stronger suppression of ΔG at higher gate voltages, where more subbands are available[8]. Although these theories consider non-interacting systems, our calculations generally show a similar behavior for systems with and without edge charge accumulation (Supplementary Fig. 7). In general, the average occupation increases faster for the non-uniform potential as the gate voltage is increased, but the larger accumulation of charge at the edges also makes these systems more susceptible to edge disorder.

Supplementary Note 4. Geometrical corrections for the quantum Hall conductance in homogeneous two-terminal graphene nanoconstrictions

The presence of a nonzero longitudinal conductivity might cause deviations of the conductance from its corresponding quantized value in two-terminal configuration in spatially uniform and homogeneous 2D conductors, depending on the device geometry.[7,18,19] Here, we exclude this possibility in our nanoconstrictions which exhibit conductance quantization suppression CQS (Sample type 1).

The geometrical correspondence between homogeneous conductors of arbitrary shape and equivalent rectangles[18,19], allows us to simplify the problem of calculating the two-terminal device conductance G in our full nanoconstriction geometry to that one of a rectangle with an aspect ratio $L/W \sim 3.8$. This is easily done by transforming the Cartesian coordinates (x, y) to elliptic coordinates (μ, ν) as follows:

$$\begin{aligned} x &= \cosh(\mu)\sin(\nu) \\ y &= \sinh(\mu)\cos(\nu) \end{aligned} \qquad (3)$$

where μ is a real number and $\nu \in [-\pi/2, \pi/2]$. Therefore, the geometry of our graphene constrictions (Supplementary Fig. 8a), having a ratio between the wider and narrower widths ~ 10 and an angle $\alpha \sim \pi/2$ in (x, y) coordinates will be contained between two rectangular boundaries $|\nu| \le \nu_0 = \pi/4$ and $|\mu| \le \mu_0 = 3$ in the (μ, ν) coordinate system. We calculate G using a general method[7,18,19] which evaluates the total current I and voltage drop V on a rectangular sample $G = I/V$. Briefly, the conductivity tensor is calculated using the semicircle relation for quantum Hall systems[7,18,19]. This relation gives a plateau-to-plateau transition between filling factors $N_1 < N_2$ (in units of e^2/h) :

$$\sigma_{xx}^2 = (\sigma_{xy} - N_1)(N_2 - \sigma_{xy}) \quad (4)$$

Landau-level (LL) broadening due to disorder is included in the calculation as a Gaussian $\sigma_{xx} = \frac{1}{2}(N_2 - N_1)e^{-\lambda(N-N_n)^2}$, where λ is a fitting parameter and N_n is the filling factor at center of the LL. The total conductivity tensor results from adding the individual filling factor dependences for each LL. Finally, the current density distribution needed to obtain I, V and G can be analytically calculated for a rectangular sample with an arbitrary aspect ratio by conformal mapping.[7,18,19]

Supplementary Fig. 8b shows the calculated G for rectangles with aspect ratios $L/W \sim 1$, $L/W \sim 2$ (similar geometry than our graphene strips, Supplementary Figs. 1 and 2) and $L/W \sim 3.8$ (equivalent geometry to our graphene nanoconstrictions).

We compare explicitly these calculations with experiments in Supplementary Figs. 8c (graphene strip $L/W \sim 2$) and 8d (graphene nanoconstriction $L/W \sim 3.8$ with rough edges, Sample type 2). The agreement with the experimental data is excellent for the LL0 in both cases. We attribute the mismatches existent at higher **B** in both samples (mainly in the nanoconstriction case, Sample type 2) to the reported mixing of non-nearest LLs at higher energies in graphene[7] and/or edge disorder.

In consequence, these calculations demonstrate that features introduced by geometrical corrections can explain some of the effects observed in the constrictions of type 2 (rough edges). However, they are not consistent with the increased conductance with suppressed quantization associated with the CQS phenomenon, observed in our constrictions of type 1 (smooth edges).

Supplementary Note 5. Further devices: Conductance at $|\mathbf{B}| \neq 0$ T in graphene nanoconstrictions with inhomogeneous charge density

Fig. 2, main text shows major differences in the conductance values of samples etched with low and higher edge roughness when $|\mathbf{B}| \neq 0$ T. The main features discussed in the main text are confirmed by further measurements over a range of samples and magnetic field strengths. The two-terminal magnetoconductance of Sample type 1 (low edge disorder) is not quantized for any of the three shown LL (see Supplementary Fig. 9a,c for $|\mathbf{B}| = 10$ T and Supplementary Fig. 10 for $|\mathbf{B}| = 6, 8, 10$ and 12 T), and shows a peak whose value is much larger than the expected quantization at the corresponding LL. This phenomenon is a clear signature of CQS[20,21] and is predicted to be absent in samples with higher edge disorder[21], as demonstrated here with our Sample type 2 devices (Supplementary Figs. 9 b,d). The latter samples exhibit a clear quantized G at $k = 0$, the LL closest to the edge of the nanoconstriction and thus most affected by edge disorder[21]. Samples with rougher edges also present a dip in G at higher V_g, in agreement with geometrical effects for homogeneous devices with $L > W$[7](Supplementary Note 4). Furthermore, we note how in Sample type 2 devices, LLs $k = 1,2$ show mixed effects from both charge density gradient and edge disorder. In particular, LLs $k = 1,2$ in Sample type 2 show a magneto-conductance reaching lower values than those expected for the single-electron picture. This behavior agrees with reported theoretical[21] and experimental studies[22], which ascribe it to disorder induced mixing of edge-currents across the entire width of the nanostructure.

Supplementary Note 6. Conductance quantization suppression in the presence of counterpropagating states: Landauer-Buttiker analysis

The CQS effect in our samples emerges due to the appearance of pairs of counter-propagating states at high magnetic field in the presence of a inhomogeneous charge density across our devices. We consider the conductance in the presence of such states[20,23] through a simple Landauer-Büttiker analysis[24]. We consider the two-terminal device shown in Supplementary Fig. 11. When the central part of the device is around the LL at filling factor N_{R}, one edge of the ribbon supports N_{R} regular edge states propagating in the same direction and the opposite edge supports an equal number of oppositely propagating states. The suppression of backscattering within the QH regime gives these states a transmission probability $T_{\mathrm{R}} = 1$. A non-uniform electron density introduces additional conducting channels by deformation of the LLs in the previously insulating bulk (Fig. 3d, main text). This generates additional N_{A} propagation channels[21,23,25]. By setting T_{A} as the transmission probability of the additional N_{A} modes, the two-terminal conductance is given as a function of T_{A}:

$$G = \frac{e^2}{h} N = \frac{e^2}{h}\left(N_{\mathrm{R}} + T_{\mathrm{A}} N_{\mathrm{A}}\right) \quad (5).$$

The conductance is quantized in the two extremal cases. For strong backscattering, $T_{\mathrm{A}} = 0$, and $G = \frac{e^2}{h} N_{\mathrm{R}}$, recovering the quantized value of a non-interacting electron system. On the other hand, in the complete absence of backscattering $T_{\mathrm{A}} = 1$, and the conductance reaches a quantized value $G = \frac{e^2}{h}\left(N_{\mathrm{R}} + N_{\mathrm{A}}\right)$, larger than that expected from the single-electron picture. In any other intermediate case ($0 < T_{\mathrm{A}} < 1$), scattering between counter-propagating pairs of the

newly introduced states exists, and removes the quantization of G . G acquires peaks whose values are larger than the expected quantization values of the single-electron picture. This is the CQS regime[21], shown in our experiments.

Furthermore, one can estimate the inhomogeneous charge density $n(x)$ needed across a narrow graphene device (ribbon) to introduce a minimum of one pair of counter-propagating edge-states in order for the CQS effect to appear[23]:

$$\frac{n(x)}{n_0} > \frac{2e^2 b|\mathbf{B}|}{\hbar \pi \varepsilon V_g} \qquad (6)$$

This approximate expression is obtained considering that the number of additional occupied LL at a point in the constriction to be[23]: $k_B(x) = \left[\frac{U(x)}{E_0}\right]^2 = Int\left[\frac{\hbar \pi n_{el}(x)}{2e|\mathbf{B}|}\right]$,

and taking into account the equality (Eq. 10, main text) $n_0 = \frac{\varepsilon V_g}{eb}$. In the former expression $U(x) = -\hbar v_F \sqrt{\pi n_{el}(x)}$ is the potential variation with respect to the Fermi level and $E_0 = \sqrt{2e\hbar v_F^2 |\mathbf{B}|}$.

Supplementary Equation 6 indicates that the increased conductance with suppressed quantization in a given nanostructure occurs from low $|\mathbf{B}|$ until the aforementioned condition is no longer valid. The exact magnetic field at which the quantization is restored will highly depend on the sample quality. Experimentally, we can confirm the existence of the CQS phenomenon at low $\mathbf{B}$. For the quality of our graphene samples, plateaus in the quantum Hall regime begin to be very clear in our large devices (width 1μm) for the LL0 at $|\mathbf{B}|$= 3T (see Supplementary Fig. 2). At $|\mathbf{B}|$=3-4 T, the CQS phenomenon in our narrow graphene constrictions with smooth edges is also seen for the LL0 (see Supplementary Fig. 15).

Supplementary Note 7. Conductance evolution from $|\mathbf{B}| = 0$ T to $|\mathbf{B}| \neq 0$ T in narrow graphene devices: literature review

In this section, we discuss and compare magneto-transport results reported in literature in high-quality and narrow graphene devices with our results. All other reported experiments show a quantized conductance at high $\mathbf{B}$ (see Supplementary Table 1). In general, the acute sensitivity of the conductance quantization suppression (CQS) phenomenon to the device electrostatics and disorder demonstrated in our findings explain the absence of this effect in the literature to date. Magnetotransport measurements in different narrow, high-quality but non-ballistic graphene devices have been reported by several groups[11,26-30]. These are high-quality devices in terms of bulk mobility and/or very low edge roughness, and even include pristine (natural) edges in some cases[27]. Importantly, independently of their measuring configuration in 2- or 4-terminal set-ups, none of these devices show a quantized conductance at $|\mathbf{B}| = 0$T, but their conductance is quantized within the quantum Hall regime. The CQS effect is absent in these devices due to its high sensitivity to the presence of moderate or higher amounts of disorder, either in the bulk or at edges[20,21].

On the other hand, high-quality and ballistic graphene constrictions have been measured in suspended devices[31] and hBN/graphene/hBN heterostructures[13]. Both types of samples show a quantized evolution of the magnetoconductance when moving from $|\mathbf{B}| = 0$ T towards $|\mathbf{B}| \neq 0$ T. In this ballistic case, the non-observation of the CQS phenomenon can be justified due to the absence of a steep enough electron density distribution across the constriction $n_{\mathrm{el}}(x)$ as indicated in Figs. 1b,c main text. The much smoother density distribution across these ballistic constrictions is underpinned by noting how the reported lever arms in these devices have a similar value[13] to the case of an infinite capacitor model.

Supplementary Note 8. Edge roughness effect on the electron density gradient across the constriction: electrostatic approach.

While disorder is the main reason for the CQS effect to not appear in non-ballistic, narrow graphene devices; a smooth $n_{\mathrm{el}}(x)$ impedes the observation of the CQS effect in ballistic samples. In this section we emphasize how these two effects are, in fact, related. In other words, higher disorder leads to a smoother $n_{\mathrm{el}}(x)$. We demonstrate this result from a simple electrostatic point of view, comparing the calculated $\frac{n_{\mathrm{el}}(x)}{n_{\mathrm{el}}(x=0)}$ of two constrictions with similar dimensions (W =100 nm, L =100 nm) with two different degrees of edge roughness: one has no edge roughness and the other one has 10 nm of edge roughness. Supplemenary Fig. S12 clearly shows how $\frac{n_{\mathrm{el}}(x)}{n_{\mathrm{el}}(x=0)}$ is much reduced in the constriction with edge roughness. In addition, we note that the effect of edge disorder should be more pronounced in experimental devices, where scattering from defects present at the edges would decrease further the effective charge accumulation at the boundaries of graphene nanostructures.

At a more general level, this result emphasizes the critical importance of quantifying the disorder in narrow, high-quality graphene devices where Coulomb interactions may need to be taken into account [23,25-27,32] or not [13,28,30,31] depending on the degree of edge disorder.

Supplementary Note 9. Introduction of random disorder in tight-binding calculations

Disordered regions of length similar to the experimental constrictions (~100 nm) are generated by removing atomic sites near the edge according to a combination of two strategies, examples of which are shown in Fig. 3b of the main text:

- An edge profile with *smooth* disorder (≤ 1 nm) follows from the superposition of 20 sinusoids with randomized start and end points, amplitudes, periods and phases. An additional envelope function ensures a continuous transition to the pristine lead regions.
- We include an additional *rough* disorder by making several sweeps $l = 1,...M$ along the edge, removing atoms randomly with probability $P_{\mathrm{r}}(l) = 0.1\left(1 - \frac{l}{M}\right)$. The results for rough disorder in Fig. 3, main text use M =50.

Supplementary Note 10. Additional tight-binding simulations of quantization suppression features.

In this section, we address some additional features of the quantization suppression effect which emerges from our simulations, and which help us to interpret the experimental data more clearly. As discussed in the text, the appearance of a CQS peak depends on a non-uniform gating potential – however if the charge neutrality point (CNP) coincides with zero gating, then no peak will appear for the 0th LL. Nevertheless, a peak feature is clearly visible in the experimental data. In Supplementary Fig. 14a, we address the possibility of zigzag edge states contributing to such a feature. With the 3rd nearest neighbor tight binding model (NNTB) such states are dispersive, and lead to an electron hole asymmetry for pristine ribbons (grey curve). However the additional hole-side channels are very quickly suppressed by even smooth edge disorder (red curve), restoring the e-h symmetry seen within the 1st NNTB model (Supplementary Fig. 14b).

Given their quick suppression by disorder, and the fact that large sections of zigzag edges are unlikely to occur in our samples, we do not consider such effects to be the cause of the LL0 peak in the experimental data. A more likely cause is an offset of the charge neutrality and zero gating points due to residual doping effects. This results in a non-uniform potential at the CNP, and the appearance of conductance peaks for LL0. This is shown in Supplementary Fig.14c for a simple model, where pristine zigzag nanoribbons are considered within the 1st NNTB model. A shift in the Fermi energy is introduced, corresponding to an additional uniform charge being added to the system, so that a finite (and thus non-uniform) gating is required to reach the CNP. We show the case for increasing values of this shift, with the red curve corresponding to the result in the main manuscript. In each case the gate voltage is corrected, and shown relative to the charge neutrality point. The emergence of a prominent peak is clear as the Fermi energy

shift is increased. In real experimental systems, the residual charging mechanisms will be more complex, and may themselves be non-uniform due to, e.g. charging of edges due to dangling bonds and trapped charges near the CNP[12,13]. Nevertheless, these mechanisms should lead to qualitatively similar features near the 0th LL. Finally, in Supplementary Fig. 14d, we demonstrate that the CQS peaks displayed by zigzag edged ribbons in the main manuscript also occur in smooth-edged armchair edged ribbons, and are suppressed by stronger edge disorder.

$G = G(V_g, \|\mathbf{B}\| = 0\text{T}) \rightarrow G = G(V_g, \|\mathbf{B}\| \neq 0\text{T})$	***Ballistic graphene device***	***Non-ballistic graphene device***
Non-interacting electrons	Quantized → Quantized<br>Refs. 13,31	Non-Quantized → Quantized<br>Refs. 26,27,30
Interacting electrons<br>***(narrow devices of W ≤ 100nm)***	A. Low edge disorder:<br>Quantized →Non-Quantized<br>*(This study, Fig. 2a, main text)*<br><br>B. Higher edge disorder:<br>Quantized (smaller step ΔG) → Quantized<br>*(This study, Fig. 2b,main text)*	Non-Quantized→ Quantized<br>Refs. 11,22,28,29

Supplementary Table 1. Conductance $G = G(V_g, |\mathbf{B}|)$ evolution from $|\mathbf{B}|$ =0 T to $|\mathbf{B}| \neq 0$ T in different reported narrow graphene devices. The unique transition demonstrated in our study corresponds to the conductance evolution "Quantized → Non-Quantized", occurring in the case of narrow ballistic devices with low edge disorder.

SUPPLEMENTARY REFERENCES

1. Gammelgaard, L., *et al*.. Graphene transport properties upon exposure to PMMA processing and heat treatments. *2D Materials*, **1**, 035005 (2014).
2. Kim, S. *et al*. Realization of a high mobility dual-gated graphene field-effect transistor with Al_2O_3 dielectric. *Appl. Phys. Lett*. **94**, 062107 (2009).
3. Dean, C., *et al.* A.F. Boron nitride substrates for high-quality graphene electronics. *Nature Nanotechnol*. **5**, 722-726 (2010).
4. Hansel, S., Lafkioti, M. & Krstic, V. Suppression of short-range scattering via hydrophobic substrates and the fractional quantum Hall effect in graphene, *PSS* **6**, 376 (2012).
5. Mayorov, A.S., *et al.* Micrometer-scale ballistic transport in encapsulated graphene at room temperature. *Nano Lett*. **11**, 2396 (2011).
6. Castro Neto, A.H., Guinea, F., Peres, N.M.R., Novoselov, K.S. & A.K. Geim, A.K. The electronic properties of graphene. *Rev. Mod. Phys*. **81**, 109 (2009).
7. Williams, J.R., Abanin, D.A., DiCarlo, L., Levitov, L.S. & Marcus, C.M. Quantum Hall conductance of two-terminal graphene devices. *Phys. Rev. B* **80**, 045408 (2009)
8. Ihnatsenka, S. & Kirczenow, G. Conductance quantization in strongly disordered graphene ribbons. *Phys. Rev. B* **80**, 201407 (2009).
9. Basu, D., Gilbert, M.J., Register, L.F. & S.K. Banerjee. Effect of edge roughness on electronic transport in graphene nanoribbon channel metal-oxide-semiconductor field-effect transistors. *Appl. Phys. Lett.* **92**, 042114 (2008)
10. Dauber, J., Terrés, B., Volk, C., Trellenkamp, S. & C. Stampfer. Reducing disorder in graphene nanoribbons by chemical edge modification. *Appl. Phys. Lett*. **104**, 083105 (2014).

11. Shen, H., Cresti, A., Escoffier, W., Shi, Y., Wang, X. & B. Raquet. Peculiar magnetotransport features of ultranarrow graphene nanoribbons under high magnetic field. *ACS Nano*, **10**, 1853-1858 (2015).

12. Simonet, P., Bischoff, D., Moser, A., Ihn, T. & Ensslin, K. Graphene nanoribbons: relevance of etching process. *J.Appl. Phys*. **117**, 184303 (2015).

13. Terrés, B., *et al*. Size quantization of Dirac fermions in graphene constrictions. *Nature Comm*. **7**, 11528-11534 (2016).

14. Fernández-Rossier, J., Palacios, J.J. & Brey, L. Electronic structure of gated graphene and graphene nanoribbons. *Phys. Rev. B* **75**, 205441 (2007).

15. Han, M.Y., Brant, J.C. & Kim, P. Electron transport in disordered graphene nanoribbons. *Phys. Rev. Lett*. **104**, 056801 (2010).

16. Asano, Y. & Bauer, G.E.W. Conductance flucturations near the ballistic-transport regime . *Phys. Rev. B* **54**, 11602 (1996).

17. Libisch, F., Rotter, S. & Burgdörfer, J. Coherent transport through graphene nanoribbons in the presence of edge disorder. *New J. Phys*. **14**, 123006 (2012).

18. Abanin, D.A. & Levitov, L.S. Conformal invariance and shape-dependent conductance of graphene samples. *Phys.Rev. B*, **78**, 035416 (2008).

19. Abanin, D.A., Skachko, I., Du, X., Andrei, E.Y. & Levitov, L.S. Fractional quantum Hall effect in suspended graphene: Transport coefficients and electron interaction strength. *Phys. Rev. B*, **81**, 115410 (2010)

20. Shylau, A.A., Zozoulenko, I.V., Xu, H. & Heinzel, T. Generic suppression of conductance quantization of interacting electrons in graphene nanoribbons in a perpendicular magnetic field. *Phys. Rev. B* **82**, 121410 (2010).

21. Shylau, A.A. & Zozoulenko, I.V. Interacting electrons in graphene nanoribbons in the lowest Landau level. *Phys. Rev. B* **84**, 075407 (2011).

22. Ribeiro, R. *et al.*. Unveiling the magnetic structure of graphene nanoribbons. *Phys. Rev. Lett*., **107**, 086601 (2011).

23. Silvestrov, P.G. & Efetov, K.B. Charge accumulation at the boundaries of a graphene strip induced by a gate voltage: Electrostatic approach. *Phys. Rev. B*, **77,** 155436 (2008).

24. Datta. S. *Electronic Transport in Mesoscopic Systems*. Cambridge University Press (1997)

25. Cui, Y-T. *et al*. Unconventional correlation between quantum Hall transport and bulk state filling in gated graphene devices. *Phys. Rev. Lett*., **117**, 18661 (2016).

26. Vera-Marun, I.J. *et al*. Quantum Hall transport as probe of capacitance profile at graphene edges. *Appl. Phys. Lett*. **102**, 013106 (2013).

27. Barraud, C., *et al*. Field effect in the quantum Hall regime of a high mobility graphene wire. *J. Appl. Phys*. **116**, 073705 (2014).

28. Hettmansperger, H., Duerr, F., Oostinga, J.B., Gould, C., Trauzettel, B. & Molenkamp L.W. Quantum Hall effect in narrow graphene ribbons. *Phys. Rev. B*. **8**, 195417 (2012).

29. Bischoff, D. *et al*. Reactive-ion-etched graphene nanoribbons on a hexagonal boron nitride substrate. A*ppl. Phys. Lett.* **101**, 203103 (2012).

30. Du, X., Skachko, I., Duerr, F., Luican A. &Andrei, E. Fractional quantum Hall effect and insulatinog phase of Dirac electrons in graphene. *Nature* **462**, 192-195 (2009).

31. Tombros, N. *et al*. Quantized conductance of a suspended graphene nanoconstriction. *Nature Phys*. **7**, 697-700 (2011).

32. Taychatanapat, T., Watanabe, K., Taniguchi, T. & Jarillo-Herrero, P. Electrically tunable transverse magnetic focusing in graphene. *Nature Phys.*, **9**, 225-229 (2013).
